# Temporal Consistency for Reliability Enhancement in Correlation-Based Time–Frequency Domain Reflectometry

**DOI:** 10.3390/s26061986

**Published:** 2026-03-22

**Authors:** Ju-Bong Lee, Hee Su Lim, Chun-Kwon Lee

**Affiliations:** 1 Department of Intelligent Robot Engineering, Pukyong National University, Busan 48513, Republic of Korea; ljb39109@gmail.com (J.-B.L.);; 2 Department of Control and Instrumentation Engineering, Pukyong National University, Busan 48513, Republic of Korea

**Keywords:** artifact suppression, reflectometry-based sensing, sensor data reliability, temporal consistency, industrial monitoring

## Abstract

Reflectometry-based sensing systems are widely used in industrial monitoring to assess the condition of distributed assets such as cables and transmission lines. In practical sensing environments, however, correlation-based interpretation can become unreliable because of bilinear interference, dispersive propagation, and excitation mismatch, often producing artifact-related responses that lead to unnecessary inspections and reduced decision reliability. This paper proposes a temporal-consistency-based reliability enhancement framework for correlation-driven time–frequency domain reflectometry (TFDR). Instead of replacing the conventional reflectometry pipeline, the proposed method introduces a reliability-estimation layer that evaluates the trustworthiness of correlation responses and suppresses temporally inconsistent artifacts. Multiple complementary descriptors extracted from the reflected signal are jointly analyzed to determine whether a correlation response is propagation-consistent or more likely to arise from non-physical artifacts. Temporal consistency is modeled using a bidirectional long short-term memory (BiLSTM) architecture that captures long-range dependencies along the propagation sequence. Experimental results obtained from cable reflectometry measurements under varying impedance conditions show that the proposed framework effectively suppresses artifact-related correlation responses while preserving physically meaningful reflections required for fault localization. Additional cross-excitation evaluation provides preliminary evidence that the learned temporal-consistency criterion is not tightly coupled to a single excitation waveform. Because the proposed framework operates as a post-processing reliability layer, it can be integrated into existing reflectometry-based monitoring systems without the modification of the sensing hardware or excitation scheme.

## 1. Introduction

Reflectometry-based sensing systems are widely used in industrial monitoring to assess the condition of distributed assets such as cables, pipelines, and transmission lines. In such environments, artifacts caused by unreliable sensor-data interpretation may lead to unnecessary inspections, increased maintenance costs, and operational downtime. Because physical access to these assets is often limited or costly, improving measurement reliability is essential for practical deployment.

Time–frequency domain reflectometry (TFDR) has emerged as an effective sensing technique for analyzing nonstationary wave propagation in distributed systems. By correlating incident signal (IS) and reflected signal (RS), TFDR enables localization of impedance discontinuities using a single sensing point, making it attractive for embedded and remote monitoring applications [1,2]. However, in practical sensing environments, the reliability of correlation-based TFDR measurements is often compromised by dispersive propagation, bilinear interference, and excitation mismatch. Bilinear time–frequency representations are known to generate cross-term interference, which can manifest as spurious correlation peaks unrelated to physical reflections [3,4,5]. In addition, dispersive propagation, attenuation, and excitation mismatch distort the reflected waveform, degrading phase coherence and further reducing the interpretability of correlation responses [6,7]. As a result, locally strong correlation values do not necessarily correspond to physically meaningful reflections, reducing the reliability of sensing decisions and increasing artifacts. Existing approaches to mitigate these issues have primarily focused on refining time–frequency representations, designing optimized excitation signals, or applying heuristic post-processing techniques such as filtering, peak thresholding, or rule-based selection. More recently, data-driven approaches based on neural networks have also been explored to suppress spurious responses [8,9,10]. However, existing neural-network-based reflectometry methods differ from the proposed framework in several important respects. Prior studies [8,9,10] typically formulate reflectometry analysis as a fault detection or classification problem, where the network directly predicts fault presence or type from reflectograms or time–frequency representations. In contrast, the proposed method formulates the task as a reliability estimation problem: instead of replacing the cross-correlation-based interpretation stage, it generates a continuous reliability-related weighting that modulates the correlation output while preserving the conventional TFDR processing chain. Moreover, whereas [10] applies a region-based convolutional neural network to two-dimensional time–frequency images, the proposed framework operates on a one-dimensional multivariate sequence of complementary descriptors (RS, NCC, NIF, and SAL), which better reflects the sequential structure of wave propagation. This representation motivates the use of a bidirectional temporal model to capture long-range dependencies. Finally, unlike most existing learning-based reflectometry approaches trained and evaluated under matched excitation conditions, this study also includes a cross-excitation experiment in which a model trained on chirp excitation is evaluated on stepped-frequency signals, providing preliminary evidence of excitation-robust temporal-consistency learning.

A key observation motivating this work is that physically meaningful reflections form temporally coherent structures, whereas artifact responses tend to appear intermittently. True reflections remain consistent across time-domain waveforms, correlation profiles, and physically interpretable descriptors derived from frequency or statistical characteristics. In contrast, bilinear artifacts and noise-induced responses tend to be transient, inconsistent, or localized to a single representation. However, explicitly modeling such consistency using deterministic signal processing rules is challenging due to the complex interaction between propagation effects, bilinear interference, and measurement noise. Therefore, assessing the reliability of correlation responses requires analyzing their temporal behavior across multiple complementary descriptors, rather than relying solely on instantaneous magnitude.

This paper addresses the above limitation by introducing a temporal-consistency-based reliability enhancement framework for correlation-driven time–frequency domain reflectometry. Rather than proposing a new reflectometry modality or replacing existing correlation-based localization methods, the proposed approach provides a reliability estimation layer that evaluates the trustworthiness of correlation responses. Multiple complementary signal descriptors are jointly analyzed to determine whether a correlation peak is propagation-consistent or more likely caused by artifacts or distortion effects. Temporal consistency is estimated using a bidirectional temporal model that exploits long-range dependencies across the measurement sequence. This design allows the framework to suppress unreliable correlation peaks while preserving physically meaningful reflections, without altering the underlying time–frequency formulation.

To clarify the scope and novelty of the proposed approach, the main contributions of this work are summarized as follows:

- A temporal-consistency-based reliability estimation framework is introduced for correlation-driven TFDR. The proposed method operates as a post-processing reliability layer that evaluates the trustworthiness of correlation responses without modifying the sensing hardware or excitation waveform.
- A multi-descriptor representation of the RS is developed by jointly integrating the RS, NCC, NIF, and SAL descriptors. These complementary features capture different physical and statistical aspects of wave propagation.
- A bidirectional temporal model is introduced to evaluate propagation-consistent patterns in reflectometry responses and suppress spurious correlation peaks caused by bilinear interference, dispersion, and excitation mismatch.
- The proposed framework is experimentally validated under both matched and cross-excitation conditions using two cable measurement scenarios, demonstrating significant artifact reduction while preserving physically meaningful reflections.

The proposed framework is evaluated using reflectometry measurements on electrical cables under varying propagation lengths and excitation conditions, including excitation types not observed during training. Experimental results demonstrate that the learned reliability weighting effectively suppresses artifacts caused by bilinear interference and excitation mismatch while maintaining the interpretability and localization capability of conventional correlation-based analysis. The proposed framework improves the reliability of reflectometry-based sensor data interpretation without modifying the sensing hardware or excitation scheme, making it suitable for integration into existing monitoring systems.

The remainder of this paper is organized as follows: Section 2 introduces the theoretical background of TFDR and temporal dependency modeling. Section 3 presents the proposed reliability enhancement framework. Section 4 presents the experimental setup and fault scenario, with results and analysis detailed in Section 5. Finally, Section 6 concludes with findings and future research directions.

## 2. Theoretical Background

### 2.1. Reliability Limitations in Correlation-Based Time–Frequency Domain Reflectometry

TFDR localizes impedance discontinuities by correlating an incident waveform with its reflected response in a joint time–frequency representation [1]. In practice, a time–frequency-modulated excitation (e.g., a Gaussian-envelope linear chirp (GELC)) is injected into the cable, and the resulting reflections are analyzed to infer spatial information along the propagation path. Let the IS be denoted by s(t) and the RS by r(t). TFDR commonly employs quadratic time–frequency representations, such as the Wigner–Ville distribution (WVD), to characterize these signals [3,4]. The similarity between the incident and reflected signals is evaluated using the NCC, which measures their local time–frequency alignment. The NCC output can be expressed as a pointwise measure evaluated independently at each time index *t* as follows:

(1) $$NCC\left(t\right)=\frac{\langle R(t,\;f),\;S(t,\;f)\rangle }{{\left||R\left(t,\;f\right)|\right|}_{2}{\left||S\left(t,\;f\right)|\right|}_{2}},$$

where R(t, f) and S(t, f) denote the time–frequency representations of the RS and IS, respectively.

Although this pointwise measure enables high-resolution localization, its reliability may degrade under non-ideal sensing conditions. Quadratic representations inevitably generate cross-term interference when multiple signal components overlap, producing correlation peaks unrelated to physical reflections [5]. In addition, analytic-signal constraints such as the Bedrosian condition and the uncertainty principle may introduce distortions that reduce the interpretability of NCC responses [11,12]. These effects are further aggravated by dispersive propagation and attenuation, which degrade phase coherence. Under such conditions, artifact-related responses may reach magnitudes comparable to genuine reflections, leading to significant decision uncertainty [13,14].

A further limitation is that conventional NCC evaluates similarity independently at each time index *t* and therefore treats correlation responses as isolated observations. Reflectometry signals, however, are generated by wave propagation along a physical medium and thus exhibit inherent temporal structure. Genuine reflections originating from impedance discontinuities tend to evolve coherently across neighboring samples, whereas artifact-related responses caused by bilinear cross-terms, noise, or excitation mismatch are often transient and locally inconsistent. Consequently, evaluating correlation magnitude alone may lead to unreliable interpretation, particularly in dispersive or mismatched sensing environments. These observations motivate the incorporation of temporal dependency modeling as a complementary mechanism for distinguishing propagation-consistent reflections from non-physical artifacts.

### 2.2. Temporal Dependency Modeling for Propagation-Consistent Interpretation

Reflectometry measurements are structured sensor responses generated by wave propagation and therefore exhibit temporal continuity along the propagation axis. Physically meaningful reflections tend to persist coherently across the measurement sequence, whereas responses caused by bilinear interference or noise are typically transient. Consequently, locally high correlation values do not necessarily correspond to physically meaningful events. Evaluating temporal consistency therefore provides a complementary criterion for improving the reliability of correlation-based interpretation. Because the measured response is a superposition of multiple delayed and dispersive components, interpretation of a given sample may depend on both preceding and subsequent observations. This motivates the use of data-driven temporal dependency modeling for reliability assessment rather than replacing analytical reflectometry.

### 2.3. Baseline Comparison

**Figure 1:**
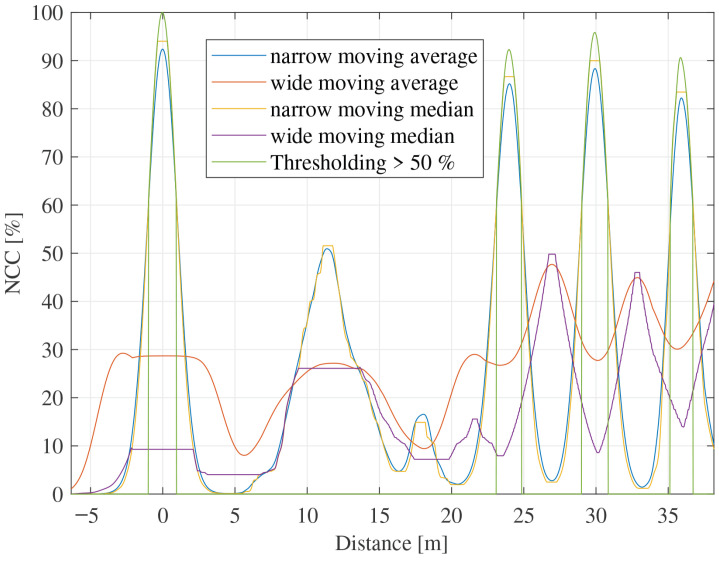
Comparison of NCC artifact suppression using representative baseline post-processing methods for a 1 kΩ fault at 24 m.

As simpler baselines, moving-average, moving-median, and fixed-threshold filtering can be applied to the NCC for artifact suppression. Figure 1 compares representative examples of these approaches. However, moving-average and moving-median filtering require a trade-off in window selection: narrow windows may distort physically meaningful peaks, whereas wide windows may leave residual artifacts insufficiently suppressed. Fixed-threshold filtering is also sensitive to threshold selection because NCC amplitudes may vary across measurements under intermittent signal-quality degradation. These limitations further motivate the use of temporal dependency modeling to distinguish propagation-consistent reflections from non-physical responses.

### 2.4. Scope of the Proposed Contribution

To clarify the boundary between the classical TFDR pipeline and the proposed framework, the following distinction is maintained throughout this paper. The classical processing chain consists of two stages: (i) the computation of the WVD of the IS and RS, and (ii) the pointwise evaluation of the NCC as defined in Equation (1). This chain constitutes the baseline interpretation mechanism and is retained without modification. The contribution of this work begins at the output of this classical pipeline. Specifically, the proposed framework introduces a post-processing reliability layer comprising three components: (i) a multi-descriptor representation of the RS that jointly integrates the RS, NCC, NIF, and SAL descriptors; (ii) a bidirectional temporal dependency model that evaluates propagation-consistent patterns across the descriptor sequence; and (iii) a reliability mask that modulates the NCC output without altering the underlying time–frequency formulation. The proposed framework therefore does not replace the physics-based TFDR processing chain but operates as a compatibility-preserving reliability layer on top of it.

## 3. Proposed Methodology

### 3.1. Overview of the Proposed Reliability Enhancement Framework

Motivated by the temporal-consistency perspective discussed in Section 2, this work proposes a reliability enhancement framework for correlation-based time–frequency domain reflectometry. The objective is to improve the interpretability of NCC responses by suppressing unreliable correlation peaks while preserving physically meaningful reflections.

Given an RS measurement, multiple complementary descriptors are extracted and jointly analyzed to determine whether a correlation response is propagation-consistent or more likely caused by interference, distortion, or noise. The resulting reliability mask is then combined with the conventional NCC output to suppress unreliable responses while preserving physically meaningful peaks. The individual components of the framework are described below.

### 3.2. Sensitivity of Correlation Responses to Excitation Mismatch

Before introducing the proposed reliability estimation mechanism, the sensitivity of correlation-based TFDR to excitation parameter variations is examined. This analysis provides empirical motivation for assessing correlation reliability beyond instantaneous correlation magnitude.

**Figure 2:**
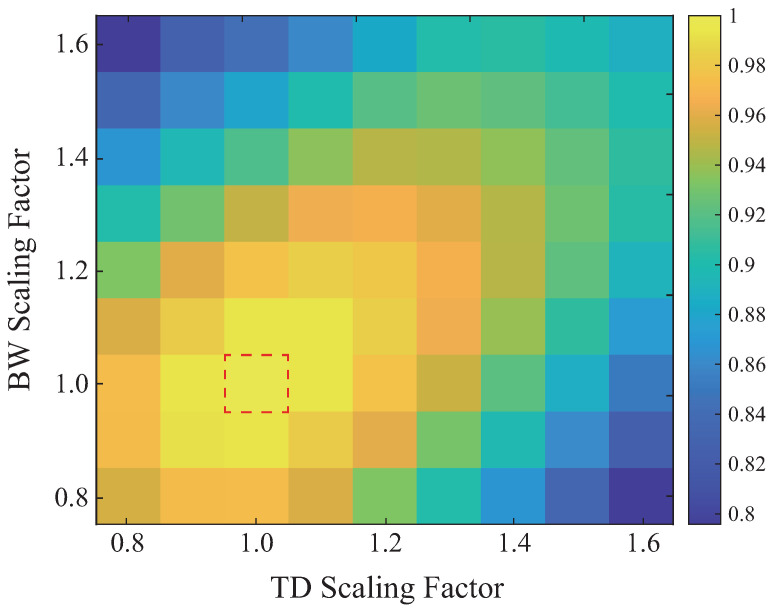
NCC sensitivity to time duration and bandwidth scaling of the GELC excitation: the box shows the NCC value when the TD and BW scale factors are 1.

The normalized cross-correlation was evaluated under controlled perturbations of excitation parameters, including TD, BW, and center frequency. Variations in TD and BW primarily affected the sharpness and localization of correlation peaks, while the peak magnitude remained relatively stable. As shown in Figure 2, independent scaling of TD and BW by up to ±60% preserved NCC peak values above 0.8, indicating that these parameters mainly influence time–frequency localization rather than spectral alignment between the IS and RS.

**Figure 3:**
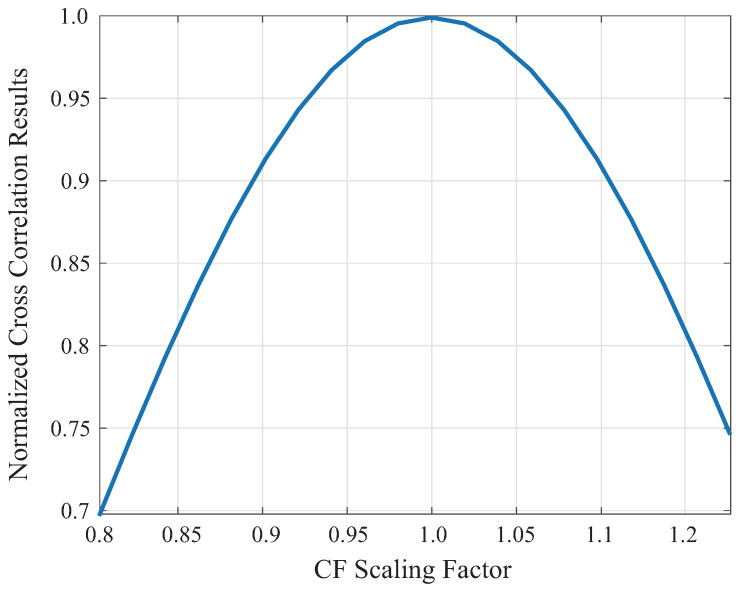
NCC sensitivity to center frequency scaling.

In contrast, deviations in center frequency produced pronounced degradation in correlation magnitude. As shown in Figure 3, when the CF deviated by more than 20% from the reference, the NCC peak dropped to approximately 0.6. Because CF governs spectral alignment and phase evolution between the IS and RS, even moderate mismatches degrade phase coherence, resulting in weakened or blurred correlation peaks.

These observations indicate that NCC responses are highly sensitive to excitation mismatch that disrupts phase coherence. Consequently, locally strong peaks may arise from factors unrelated to genuine impedance discontinuities. Correlation magnitude alone is therefore insufficient as a reliability indicator, motivating the use of additional contextual information to assess temporal consistency.

### 3.3. Temporal Consistency-Based Reliability Estimation Framework

The sensitivity analysis in Section 3.2 showed that NCC magnitude alone cannot fully distinguish genuine reflections from spurious correlations caused by cross-terms, dispersion, or excitation mismatch. To improve reliability, temporal dependency modeling is introduced using a bidirectional long short-term memory (BiLSTM) architecture, referred to here as a temporal memory unit (TMU). This choice is motivated by the long-range dependencies of reflectometry responses along the propagation axis. By integrating multiple complementary descriptors derived from the RS, the model distinguishes propagation-consistent temporal patterns from non-physical artifacts.

#### 3.3.1. Multi-Descriptor Representation of RS

To enable reliability assessment based on temporal consistency, the proposed framework integrates multiple complementary descriptors derived from the RS. Each descriptor captures a different aspect of signal behavior and provides information complementary to the NCC.

(1) Time-domain RS

The time-domain RS x[n] preserves raw amplitude and phase information associated with impedance discontinuities and reflects propagation effects such as attenuation, dispersion, and multipath interference. However, because it is sensitive to noise and distortion, it is used jointly with derived descriptors rather than as a standalone reliability indicator.

(2) Normalized cross-correlation

The NCC measures local time–frequency similarity between the incident and reflected signals and serves as the primary indicator of potential reflection events. Although it highlights matched structures, it is also susceptible to spurious responses caused by bilinear interference and excitation mismatch. In the proposed network, NCC is treated as an informative but unreliable descriptor whose trustworthiness must be evaluated in context.

(3) Normalized instantaneous frequency

The NIF characterizes the local frequency modulation behavior of the RS [15,16]. Because bilinear cross-terms do not follow physically meaningful frequency trajectories, NIF helps distinguish propagation-consistent reflections from artifacts. Although instantaneous-frequency estimates may be noisy, their temporal trends remain informative when analyzed jointly with other descriptors. For a discrete time signal $x\left[n\right]\;=\;A\left[n\right]e^{j\phi [n]}$, with amplitude A[n] and phase ϕ[n], a central-difference IF estimate ${\hat{f}}_{n}$ can be written as follows [15]:

(2) $$\hat{f}\left[n\right]\;=\;\frac{\hat{\omega }\left[n\right]}{2\pi }\;=\;\frac{\;\phi [n+1]-\phi [n-1]}{4\pi }.$$

and is normalized as

(3) $$NIF\left[n\right]\;=\;\frac{\hat{f}\left[n\right]-{\hat{f}}_{c}+\frac{BW}{2}}{BW},$$

where ${\hat{f}}_{c}$ denotes the central frequency of $\hat{f}\left[n\right]$ and BW is the effective BW of the IS. To reduce noise amplification due to differentiation, ϕ[n] or $\hat{f}\left[n\right]$ is processed via a hierarchical IF estimator [17] prior to normalization.

(4) Statistical salience descriptor

To identify signal segments that deviate significantly from the background, we employ a SAL descriptor based on false-discovery-rate (FDR) control [18,19,20]. This descriptor emphasizes localized transitions unlikely to arise from noise or background drift. Its computation proceeds through four stages:

Step 1: Derivative Computation First- and second-order finite differences, $\Delta_{l}x\left[n\right]$ and $\Delta_{l}^{2}x\left[n\right]$, are computed to represent the discrete slope and curvature of the reflected waveform. These terms capture the structural transitions and peak-like formations inherent in wave propagation.

Step 2: Robust Normalization To ensure comparability across heterogeneous cable segments, the derivatives are normalized using median-based robust estimators as follows:

(4) $$D\left[n\right]=\sqrt{{\left(\frac{\Delta_{l}x\left[n\right]}{3\sigma_{\Delta }}\right)}^{2}+0.5{\left(\frac{\Delta_{l}^{2}x\left[n\right]}{3\sigma_{\Delta^{2}}}\right)}^{2}}$$

where $\sigma_{\Delta }$ and $\sigma_{\Delta^{2}}$ denote the median absolute deviation (MAD) of $\Delta_{l}x\left[n\right]$ and $\Delta_{l}^{2}x\left[n\right]$ within a sliding window, respectively. This robust scaling ensures that the descriptor D[n] is resistant to impulsive noise and baseline drift [21,22].

Step 3: Significance Weighting The descriptor is standardized to a robust Z-score, $Z\left[n\right]=\left(D\left[n\right]-\hat{\mu }\right)/\hat{\sigma }$, which is then converted to *p*-values [23]. To control the global error rate under multiple testing conditions, Storey’s FDR procedure [19] is applied. The resulting *q*-values, representing the minimal FDR level at which a sample is significant, are used to define the final weight:

(5) SAL[n]=max0,1−q[n]/α

where α is the target significance level. This process yields a probabilistic attention signal that emphasizes meaningful reflections while suppressing stochastic fluctuations [24,25].

#### 3.3.2. Feature Integration and Preprocessing

To leverage the complementary nature of the extracted descriptors, RS, NCC, NIF, and SAL are aligned to the sampling grid of x[n] and normalized on a sequence basis. The resulting four-dimensional multivariate sequence preserves intra-feature dynamics and cross-feature relationships relevant to temporal co-occurrence. In particular, NIF provides frequency-trajectory information and SAL provides statistical significance, enabling the model to distinguish physical reflections from transient artifacts. This integrated sequence is used as the input to the temporal model.

#### 3.3.3. Reliability Estimation and Post-Enhancement

The proposed framework employs a bidirectional temporal model to estimate a learned reliability-gating signal R(t) for each time index. Rather than producing categorical decisions, the model evaluates whether an NCC response is supported by coherent behavior across multiple descriptors. High values of R(t) are associated with propagation-consistent reflections, whereas low values are associated with transient responses more likely caused by bilinear interference or distortion.

To account for the non-local nature of reflectometric signals, temporal dependencies in both directions are considered, enabling robust reliability assessment under dispersive propagation and overlapping reflections. The learned reliability-gating signal is used to modulate the NCC output multiplicatively:

(6) $$\hat{\rho }\left(t\right)=R\left(t\right)\cdot \rho \left(t\right)$$

where $\hat{\rho }\left(t\right)$ denotes the enhanced response. Because this operation is performed on the correlation output, the underlying time–frequency formulation and localization mechanism remain intact, ensuring compatibility with existing TFDR systems. This formulation emphasizes reliability modulation of the correlation profile rather than explicit peak detection.

**Figure 4:**
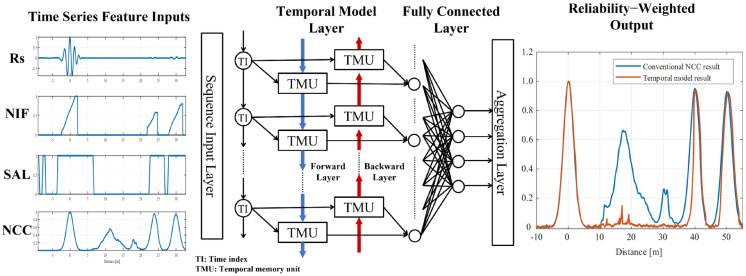
Overview of the proposed temporal-consistency-based reliability enhancement framework.

During training, reference masks are constructed to represent propagation-consistent regions. These masks guide the model to focus on consistency of signal behavior rather than explicit fault classification or location encoding. As a result, the proposed framework generalizes across varying propagation conditions and sensing configurations, providing a robust reliability layer that complements conventional correlation-based analysis. Figure 4 summarizes the overall framework. The input signal undergoes a series of processing steps before being fed into a bidirectional LSTM (BiLSTM) to capture temporal dependencies. The blue downward arrows denote the forward pass, while the red upward arrows indicate the backward pass of the BiLSTM. The final output is compared with the conventional NCC result.

The learned mask is used directly as a reliability-gating signal for NCC modulation rather than as a separately calibrated confidence score. Accordingly, practical interpretation is based on whether a given temporal region is retained or suppressed by the mask, rather than on an additional threshold selected within the proposed framework itself.

#### 3.3.4. Ablation Study of Feature Components

**Figure 5:**
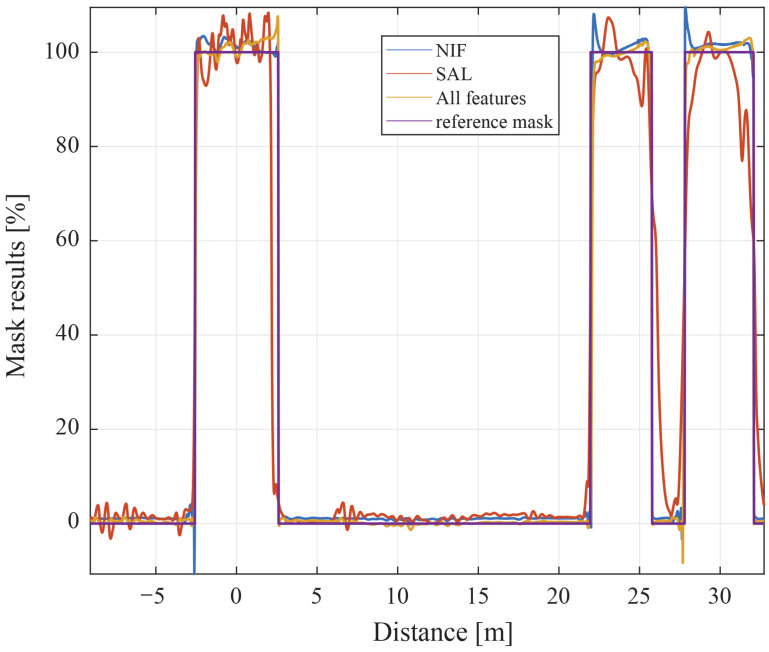
Ablation on a 30 m, 2-core XLPE cable with a 1 kΩ fault at 24 m, $Z_{0}=50\;\Omega$: reliability mask vs. temporal model estimates using RS + NIF + NCC, RS + SAL + NCC, and All features.

To quantify the contribution of each descriptor, the model was trained with various feature subsets while maintaining identical training configurations. The following analyses use the same experimental configuration as Scenario 1 in Section 4. As illustrated in Figure 5, models employing only a single auxiliary feature (e.g., RS + NIF + NCC or RS + SAL + NCC) exhibit performance degradation. Specifically, RS + SAL + NCC tends to underestimate the reliability mask, while RS + NIF + NCC leaves residual overestimation near artifact-prone regions.

In contrast, the full-feature model (All features) closely tracks the reference mask, effectively suppressing spurious responses with the lowest error (RMSE of 2.05%, see Table 1). These results confirm that no single feature fully captures the propagation and bilinear-interference characteristics of TFDR. The integration of all four descriptors is therefore essential for achieving accurate and stable reliability enhancement, particularly in the presence of dispersion and background fluctuations.

#### 3.3.5. Comparison with Alternative Network Architectures and Sequence Settings

To assess the suitability of the proposed BiLSTM architecture, alternative network backbones were compared under the same feature configuration and training setting.

**Figure 6:**
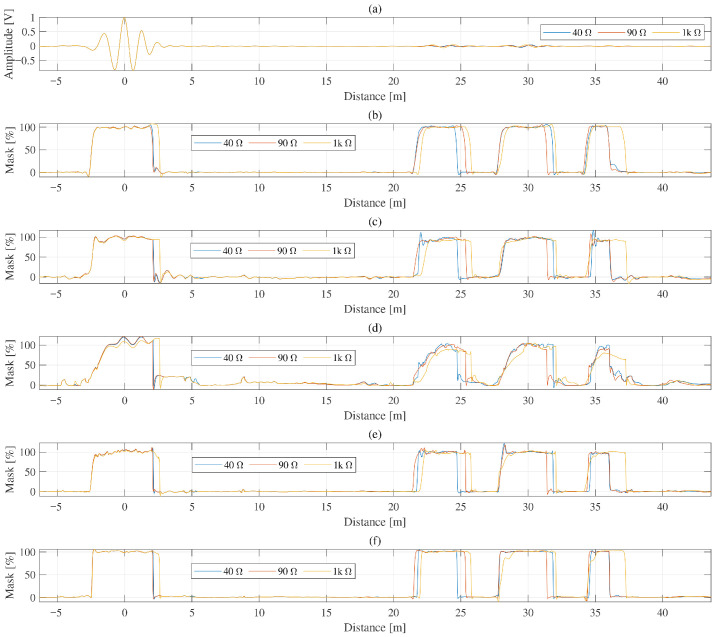
Comparison of BiLSTM and other networks with 40 Ω, 90 Ω and 1 kΩ: (**a**) measured signal, mask estimation via (**b**) BiLSTM, (**c**) LSTM, (**d**) GRU, (**e**) 1D-CNN and (**f**) recurrent CNN.

Figure 6 compares reliability-mask estimation results obtained using BiLSTM, LSTM, a gated recurrent unit (GRU), a one-dimensional convolutional neural network (1D-CNN), and a recurrent CNN, all trained on the full sequence. All models identified the major reflection regions, including the signal injection point, the fault location, and the cable-end reflection. However, noticeable differences were observed in mask stability. In particular, the LSTM, the GRU, and, to a lesser extent, the 1D-CNN outputs showed intermittent spurious activations in artifact-prone regions, whereas the BiLSTM output exhibited fewer such artifacts and a more stable mask profile.

The same tendency is reflected in Table 2. BiLSTM yielded the lowest average artifact-related RMSE among the recurrent models while maintaining a comparatively low worst-case artifact level. Although the 1D-CNN provided competitive average performance, its worst-case artifact level was higher than that of BiLSTM. These results indicate that BiLSTM provides a favorable balance between mask stability and conservative suppression of spurious activations.

Table 3 further compares BiLSTM and recurrent CNN, the strongest hybrid models considered in this study. Although the two models exhibited similar mask-estimation accuracy, BiLSTM required fewer learnable parameters and achieved shorter inference time per sequence. These results indicate that BiLSTM provides a more efficient and stable backbone for the proposed reliability-enhancement stage. In addition, a control experiment comparing whole-sequence and sliding-window processing was conducted under the same sequence-wise normalization scheme to assess dependence on full-sequence global context.

**Figure 7:**
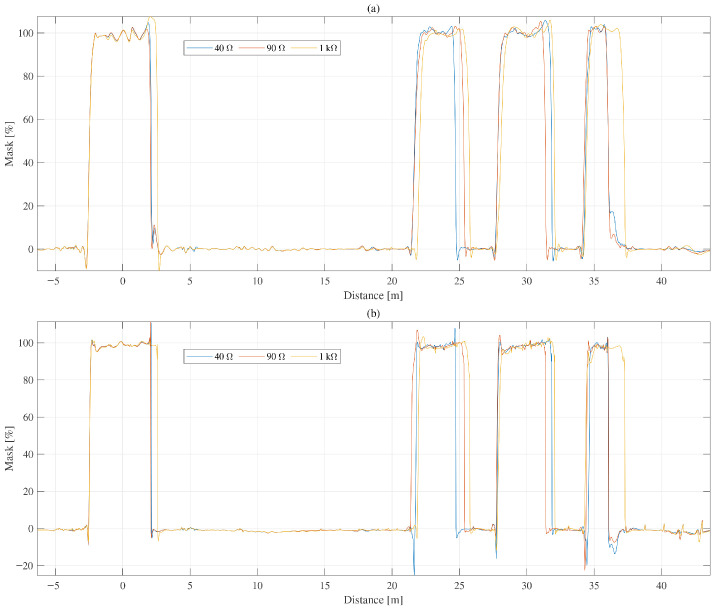
Representative comparison of reliability-mask estimation obtained from BiLSTM trained with (**a**) whole-sequence input and (**b**) sliding-window input under the same sequence-wise normalization.

Figure 7 shows representative reliability-mask estimates obtained from BiLSTM trained with whole-sequence input and sliding-window input, respectively. In both cases, the major physically meaningful reflection regions were preserved. The quantitative comparison in Table 4 shows that the sliding-window setting led to moderate degradation in reliability-mask RMSE, artifact RMSE, and worst-case artifact level, while maintaining qualitatively similar overall mask behavior. These results indicate that the proposed model is not dependent exclusively on full-sequence context and that locally persistent temporal-consistency patterns contribute materially to the learned mask behavior.

## 4. Experimental Setup

**Figure 8:**
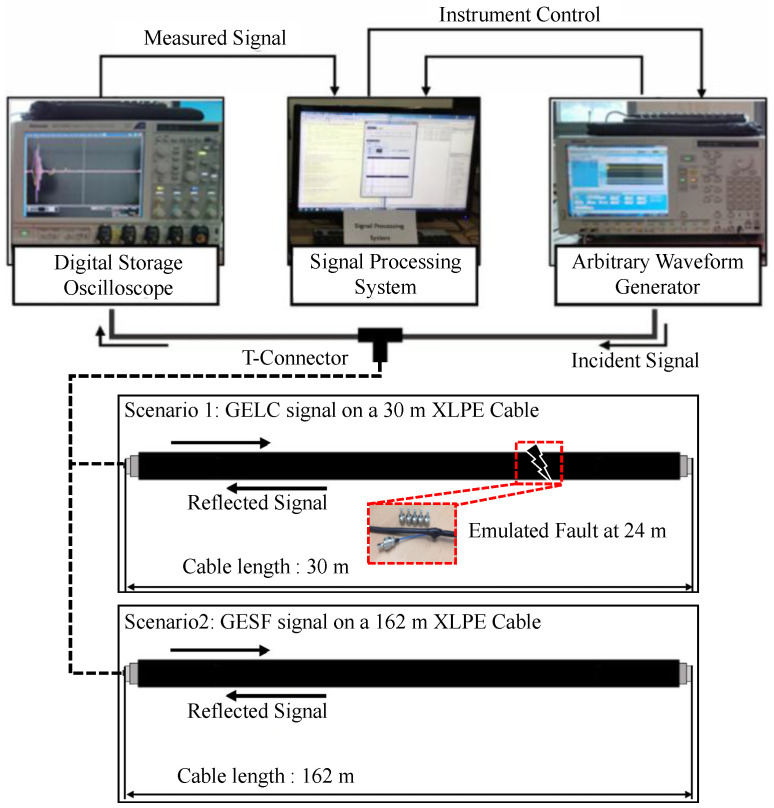
Experimental Setup for Signal Acquisition.

The proposed model was validated using two measurement scenarios. As shown in Figure 8, the measurement system consisted of an arbitrary waveform generator (AWG) and a digital storage oscilloscope (DSO), connected to the cable under test via a T-connector for single-ended reflectometry. The excitation signal was generated digitally and injected into one conductor of the two-core XLPE cable, while the RS was captured on the coupled port of the T-connector and digitized for offline processing. Faults were emulated by inserting carbon-film resistors (±1% tolerance) in series with one conductor at the designated fault location. The resistors were connected via BNC connector to ensure stable and repeatable contact impedance across repeated measurements. Scenario 1 evaluates fault detection performance under standard TFDR conditions using GELC excitation on a 30 m control and instrumentation (C&I) cable. Scenario 2 examines cross-excitation generalization using Gaussian-envelope stepped-frequency (GESF) signals on a 162 m cable. Together, these scenarios assess accuracy under matched TFDR conditions and robustness to excitation-type and propagation characteristics.

### 4.1. Scenario 1: GELC Signal on a 30 m XLPE Cable

A 30 m, two-core XLPE insulated C&I cable was used to evaluate fault detection under conventional TFDR excitation. The GELC excitation parameters were set to a center frequency of 60 MHz, bandwidth of 110 MHz, and time duration of 100 ns. Faults were emulated at approximately 24 m by inserting a series resistor; the 0 Ω level was realized by replacing the resistor with a short wire to represent a near-normal condition. Thirteen impedance levels were tested from 0 Ω to 100 kΩ (near-open-circuit), spanning resistive fault conditions across the full practically relevant range. For detailed case-wise evaluation, three impedance conditions (40 Ω, 90 Ω, and 1 kΩ) were selected as representative validation examples spanning low-, medium-, and high-impedance responses. In addition, a formal leave-one-group-out cross-validation was conducted across all 13 impedance levels to assess robustness over the full impedance range. This configuration allows evaluation of the model behavior under impedance conditions not directly observed during training. For each RS, four features were extracted: (i) NCC using the TFDR formulation of Section 3, (ii) NIF, (iii) SAL, and (iv) the time-domain RS. These features form the multivariate input to the temporal model. Scenario 1 is therefore a controlled evaluation of the model’s ability to suppress cross-terms and detect resistive fault reflections.

### 4.2. Scenario 2: GESF Signal on a 162 m XLPE Cable

Generalization across excitation types and propagation distances was examined using a 162 m two-core XLPE coaxial cable excited by GESF signals. This assesses whether a model trained solely on GELC data can operate under non-chirp excitation, as may occur in practice when the deployment hardware differs from that used during training. A rectangular window would produce sidelobes in the frequency-to-time reconstruction; therefore each tone was Gaussian-windowed. The excitation comprised tones from 5 to 32.5 MHz in 2.5 MHz steps, each with a 500 ns burst. NCC and NIF were computed using the same TFDR parameters as Scenario 1, without applying any chirp-specific assumptions.

### 4.3. Training Protocol and Construction of Reference Reliability Masks

The temporal model is trained to estimate the temporal consistency of correlation responses rather than to directly infer fault locations. Accordingly, the supervision strategy is designed to guide the model toward distinguishing propagation-consistent and inconsistent correlation behavior, instead of encoding explicit diagnostic labels.

Training is performed using full-length reflectometry sequences to preserve the global propagation context. All descriptor streams are temporally aligned along the propagation axis and normalized on a per-sequence basis to reduce sensitivity to absolute amplitude variations. The model was implemented in MATLAB R2025 using the Deep Learning Toolbox. The key training parameters are as follows: max epochs = 50, gradient threshold = 1, initial learning rate = 0.01, learning-rate schedule = piecewise (drop factor = 0.2, drop period = 50 epochs), mini-batch size = 50, and shuffle = every-epoch. The network parameters are optimized using the Adam optimizer. The output layer uses MATLAB’s regressionLayer, which corresponds to a half-mean-squared-error objective. It is also consistent with the framework’s design intent, since the network output is used as a learned reliability-gating signal for NCC modulation rather than as a hard classifier. Repeated reflectometry measurements were performed under identical experimental conditions in order to capture the variability in correlation responses caused by noise, bilinear interference, and excitation mismatch. These repeated observations provide the basis for identifying propagation-consistent reflection regions used in the construction of the reference reliability masks.

Reference masks for training are constructed through a rule-based procedure applied to repeated reflectometry measurements acquired under identical experimental conditions. Because a directly measured ground-truth segmentation is not available in the present reflectometry setting, the resulting masks are used as reference supervisory masks rather than as exact ground-truth labels.

**Step 1—Baseline characterization.** For each cable configuration, the fault-free NCC profile is first obtained as

(7) $$\rho_{0}\left(t\right)=\mathrm{NCC}\left(t\right){|}_{\mathrm{fault}\text{-}\mathrm{free}},$$

which captures the stable background reflection structure, including the injection interface and cable-end reflection. The fault-free baseline is used as a physical reference for distinguishing reproducible background responses from fault-related changes, rather than as a direct thresholding term in the subsequent IF-consistency rule.

**Step 2—Candidate-region selection from IF-consistent reflected response.** Around the physically expected fault-reflection location, a candidate region is identified from the interval in which the reflected signal maintains locally consistent IF behavior relative to the expected GELC chirp trajectory:

(8) $$R\left(t\right)=\left\{t:\left|\mathrm{NIF}\left(t\right)-{\mathrm{NIF}}_{\mathrm{ref}}\left(t\right)\right|\leq \epsilon \left(t\right)\right\},$$

where ${\mathrm{NIF}}_{\mathrm{ref}}\left(t\right)$ denotes the normalized linear chirp reference and ϵ(t) is treated as an adaptive local tolerance rather than a single fixed global threshold. In this study, ϵ(t) was scaled by a factor of 2.576 following the uncertainty formulation of the hierarchical ridge-based IF estimator [17], corresponding approximately to a 99% confidence interval for the locally estimated IF behavior. Because the excitations considered here are Gaussian-windowed, the most stable reflected-signal energy is concentrated near the central portion of the response, whereas the outer portions are more susceptible to dispersion, leakage, and artifact contamination. Accordingly, the accepted mask width was restricted to approximately 50% of the measured reflected-signal duration and used as a reproducible operational rule rather than a manually optimized threshold.

**Step 3—Persistence filtering.** To further ensure propagation consistency, only candidate regions that recur across repeated measurements under the same acquisition condition are retained, whereas non-recurring regions are discarded as unreliable artifacts.

**Step 4—Binary reference-mask generation.** The final reference mask is defined as

(9) $$M_{\mathrm{ref}}\left(t\right)=\left\{\begin{array}{cc} 1, & t\in R(t)\;\mathrm{and}\;\mathrm{recurrent}\;\mathrm{across}\;\mathrm{repeated}\;\mathrm{measurements},\\ 0, & \mathrm{otherwise}. \end{array}\right.$$

These masks do not encode fault categories or exact fault locations. Instead, they provide coarse temporal guidance indicating whether a given correlation response is likely to be supported by physically meaningful propagation behavior.

During inference, the proposed framework operates without access to fault-free baselines or reference masks. Instead, the trained model estimates reliability directly from the joint temporal behavior of the descriptor sequence and suppresses artifact-related correlation responses through the learned masking operation.

## 5. Results and Discussion

Experimental results evaluate the effectiveness of the proposed reliability-enhancement framework under different sensing conditions. In this paper, artifacts refer to temporally inconsistent NCC responses that do not correspond to physically meaningful reflections such as the signal injection point, the fault location, or the cable termination. These responses typically arise from bilinear cross-term interference, dispersive propagation, or excitation mismatch. The proposed reliability mask, $\hat{\rho }\left(t\right)=R\left(t\right)\rho \left(t\right)$, attenuates such components by modulating the NCC profile rather than performing explicit peak detection. The following analysis therefore focuses on suppressing unreliable correlation components while preserving physically meaningful reflections.

### 5.1. Scenario 1: 30 m XLPE Cable with GELC Excitation

**Figure 9:**
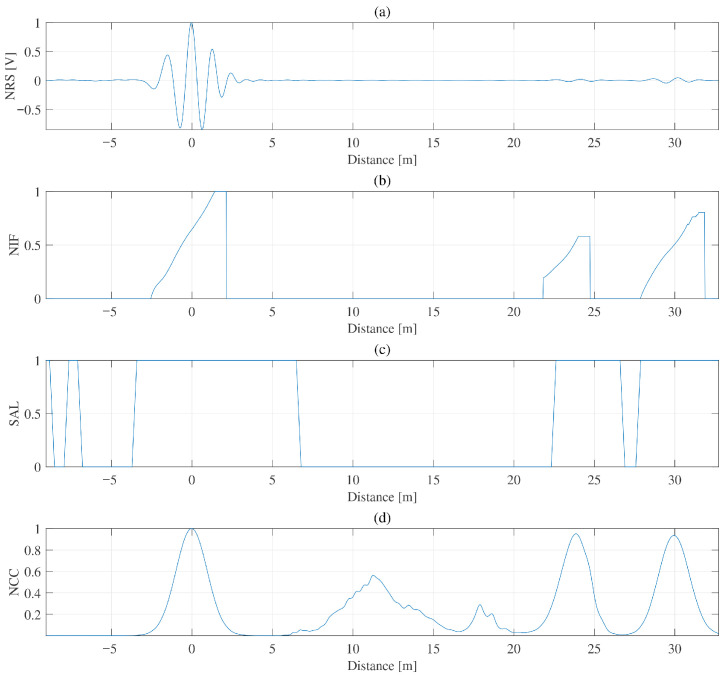
For 30 m, 2-core XLPE cable with 1 kΩ fault at 24 m, input features of (**a**) RS, (**b**) NIF, (**c**) SAL, and (**d**) NCC.

**Figure 10:**
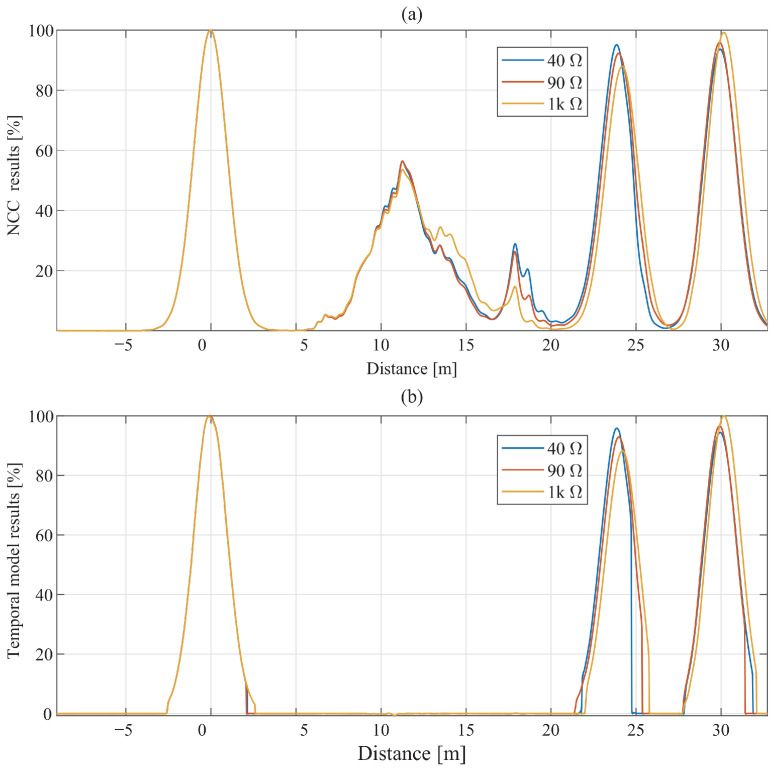
Comparison between (**a**) a conventional NCC and (**b**) the proposed model outputs for 40 Ω, 90 Ω, and 1 kΩ fault at 24 m.

Figure 9 presents the four extracted descriptors for a 1 kΩ fault condition. The NCC exhibits several spurious peaks in the range of approximately 5–20 m due to cross-term interference. The NIF reflects dispersion-related instantaneous-frequency variation but also shows isolated outliers at intermediate distances. The SAL descriptor highlights statistically significant regions, although slightly broadened intervals and occasional pre-incident artifacts are observed. These observations confirm that no single descriptor alone is sufficient to reliably distinguish physically meaningful reflections from artifact-related responses. Figure 10 presents representative validation examples for three impedance levels (40 Ω, 90 Ω, and 1 kΩ), while Table 5 summarizes the corresponding quantitative results. In all three cases, the proposed mask suppresses artifact-related NCC components while preserving the physically meaningful reflections associated with the incident, fault, and cable-end locations. The framework therefore achieves strong artifact suppression while maintaining low RMSE in the reflection regions.

To assess robustness across the full impedance range, leave-one-group-out cross-validation was performed across all 13 impedance levels, with one level used for testing and the remaining levels used for training in each fold. The corresponding results are summarized in Table 6. On average, the proposed method reduced the peak artifact level from 56.35% to 1.31% while limiting the associated scale distortion to within ±1.99%. This result indicates stable artifact suppression across the full impedance range considered in this study.

### 5.2. Scenario 2: 162 m XLPE Cable with GESF Signal

Scenario 2 examines the robustness of the proposed framework to changes in excitation type. If the model relied mainly on excitation-specific patterns learned under GELC conditions, its performance would degrade under stepped-frequency excitation. Successful artifact suppression under GESF excitation would therefore indicate that the learned criterion depends on propagation-consistent descriptor behavior rather than a specific waveform shape.

**Figure 11:**
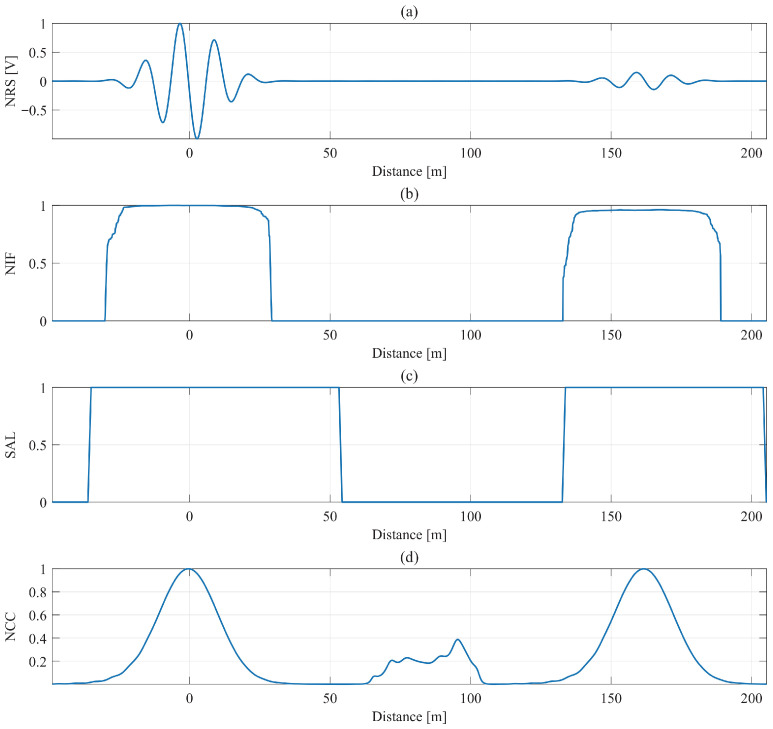
For 162 m, 2-core XLPE normal cable, input features of (**a**) GESF signal, (**b**) NIF, (**c**) SAL, and (**d**) NCC.

Figure 11 shows the extracted descriptors for the 162 m cable under GESF excitation. All descriptors correctly identify the incident and cable-end reflections expected for a healthy cable. Outside these regions, NIF, SAL, and NCC exhibit irregular fluctuations due to the stepped-frequency structure and long-range dispersion, indicating a domain shift relative to Scenario 1.

**Figure 12:**
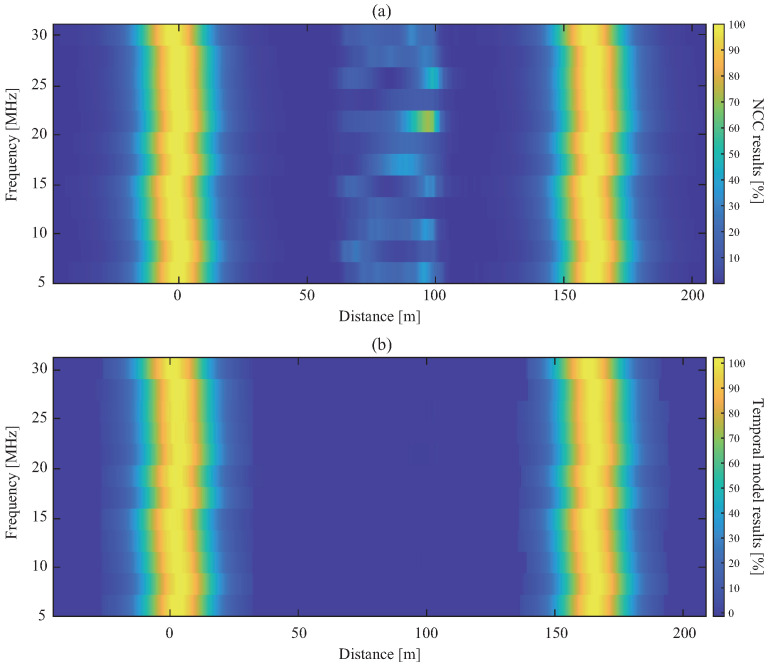
Comparison of (**a**) NCC and (**b**) temporal model results for the 162 m cable under GESF excitation.

Figure 12 compares the raw NCC and the reliability-enhanced output. The conventional NCC contains numerous intermediate peaks that could be misinterpreted as reflections, whereas the proposed model suppresses most of these components and retains only the incident and end reflections, consistent with the expected behavior of an intact cable. A small overshoot remains near the incident region, likely due to the distribution mismatch between GELC-trained feature statistics and GESF-derived descriptors. However, this residual error remains spatially localized and small (RMSE < 2.72%), and therefore does not hinder practical interpretation. Across all GESF test cases, the proposed framework achieved an average artifact reduction of 99.53%, comparable to Scenario 1 despite the signal-type shift.

These results do not constitute a comprehensive demonstration of excitation-agnostic generalization but provide preliminary evidence that the proposed temporal-consistency criterion is not tightly coupled to a single excitation waveform. Additional validation across different cable types, excitation bandwidths, and environmental conditions will be required to establish broader generalization.

### 5.3. Scope of Applicability and Limitations

Although the proposed framework improves the reliability of NCC-based interpretation, its performance remains dependent on the temporal resolution and signal quality of the underlying TFDR hardware. The present model was developed for coaxial and XLPE cable measurements in which wave propagation follows a predominantly deterministic propagation behavior. Under extremely low signal-to-noise ratios, highly branched networks, or propagation conditions that differ substantially from those represented in the training data, the reliability mask may exhibit reduced sensitivity. Because the framework operates as a modular post-processing layer, however, it can in principle be re-trained or fine-tuned for different sensing configurations without altering the underlying reflectometry pipeline.

The experimental evaluation presented in this study focused on single-fault configurations, in which one impedance discontinuity was inserted at a fixed location. In practical cable networks, multiple faults may coexist along the same line. When the inter-fault spacing exceeds the spatial resolution of the excitation signal—approximately 0.9 m for the GELC parameters used here (CF = 60 MHz, BW = 110 MHz)—the corresponding reflections may remain separable in the NCC response. In such cases, the proposed framework may still be applicable in principle, since each reflection can be associated with a locally coherent descriptor pattern. A more challenging case arises when nearby faults produce overlapping or compound correlation peaks. In such situations, the bidirectional temporal model may provide partial contextual information from both preceding and subsequent samples, although this has not been experimentally verified in the present study.

## 6. Conclusions

This paper proposed a temporal-consistency-based reliability enhancement framework for correlation-driven time–frequency domain reflectometry. The method introduces a reliability estimation layer that evaluates the temporal consistency of multiple descriptors derived from the reflected signal. By modeling long-range dependencies using a bidirectional temporal architecture, the framework suppresses unreliable correlation responses caused by bilinear interference, dispersive propagation, and excitation mismatch while preserving physically meaningful reflections associated with impedance discontinuities. Experimental results from cable reflectometry measurements demonstrated that the proposed reliability weighting effectively suppresses artifact-related correlation responses and improves the interpretability of NCC-based analysis. Across a wide range of impedance conditions, temporally inconsistent correlation components were significantly reduced without distorting physically meaningful reflection peaks.

Several limitations should be noted. First, the present validation focused on resistive impedance discontinuities at a single fault location. Although the proposed framework is designed as a reliability-enhancement layer rather than a fault-type classifier, additional experiments involving other defect types (e.g., capacitive degradation or shield discontinuities) are required to establish broader applicability. Second, the training data mainly consisted of single-fault configurations, and model behavior under multi-fault conditions has not yet been verified. Third, further validation under varying cable geometries, excitation bandwidths, noise levels, and long-term operating conditions is necessary to assess the full generalization capability of the framework. Because normalization remained sequence-wise in the present study, stricter train-global or cross-normalization controls should be examined in future work.

Overall, the proposed framework improves the robustness and reliability of correlation-based reflectometry interpretation while preserving the physics-based structure of the TFDR processing chain. Because it operates as a post-processing reliability layer, it can be integrated into existing reflectometry-based monitoring systems without modifying the sensing hardware or excitation waveform. The learned output functions directly as a reliability-gating mask, eliminating the need for an additional threshold-tuning stage. Future work will extend the framework to more diverse fault types, multi-fault scenarios, and larger datasets and will also examine alternative temporal architectures such as temporal convolutional and attention-based models.

## Figures and Tables

**Table 1:** RMSE of estimated reliability masks for different feature combinations.

Feature Set	RS + NIF + NCC	RS + SAL + NCC	All Features
RMSE [%]	3.84	11.68	2.05

**Table 2:** Comparison of artifact-related mask-estimation errors for different network backbones.

Results	BiLSTM	LSTM	GRU	1D-CNN	Recurrent CNN
Average artifact RMSE	1.00%	5.25%	15.03%	1.94%	2.86%
Worst-case artifact level	1.86%	5.39%	21.09%	5.96%	4.09%

**Table 3:** Detailed comparison between BiLSTM and the recurrent convolutional neural network.

	BiLSTM	Recurrent CNN
Learnable Parameters	22,101	39,253
Runtime per single sequence [ms]	1.05	1.34
Mask estimation RMSE	1.41%	1.60%

**Table 4:** Comparison between whole-sequence and sliding-window BiLSTM under the same sequence-wise normalization scheme.

	Average Reliability-Mask RMSE	Average Artifact RMSE	Worst-Case Artifact
Whole sequence	1.41%	1.00%	1.86%
Sliding window	1.90%	2.53%	3.41%

**Table 5:** RMSE of proposed model outputs for incident, fault, and end-reflection regions.

Results	IS Distortion [%]	Fault Distortion [%]	RS Distortion [%]	Artifact Reduction [%]
40 Ω	1.41	0.9	1.32	99.91
90 Ω	1.37	0.79	1.12	99.89
1 kΩ	1.40	1.49	1.11	99.90

**Table 6:** Levelwise Comparison of BiLSTM-Masked NCC in Leave-One-Group-Out Cross-Validation.

Resistance Value	Peak Artifact Level Before/After [%]	Overall Scale Change [%]
0 Ω	61.46/2.18	+0.64
10 Ω	58.18/0.87	−3.82
20 Ω	58.31/1.03	+3.83
30 Ω	55.24/0.57	+2.79
40 Ω	56.39/0.54	+0.39
50 Ω	56.54/0.09	+0.32
70 Ω	54.25/0.54	−0.35
80 Ω	56.20/2.13	−0.54
90 Ω	56.44/1.24	+2.93
100 Ω	53.64/2.01	−3.52
1 kΩ	54.77/1.92	+5.09
10 kΩ	57.38/1.47	+0.71
100 kΩ	53.72/2.48	−1.06

## Data Availability

The original contributions presented in this study are included in the article. Further inquiries can be directed to the corresponding author.
